# Dopamine D1‐receptor‐expressing pathway from the nucleus accumbens to ventral pallidum‐mediated sevoflurane anesthesia in mice

**DOI:** 10.1111/cns.14267

**Published:** 2023-05-19

**Authors:** Jie Zhang, Yiting Peng, Chengxi Liu, Yu Zhang, Xiaoli Liang, Chengdong Yuan, Wenyan Shi, Yi Zhang

**Affiliations:** ^1^ Department of Anesthesiology The Second Affiliated Hospital of Zunyi Medical University Zunyi China; ^2^ Guizhou Key Laboratory of Anesthesia and Organ Protection Zunyi Medical University Zunyi China; ^3^ School of Anesthesiology Zunyi Medical University Zunyi China; ^4^ Department of Anesthesiology The Affiliated Hospital of Zunyi Medical University Zunyi China

**Keywords:** general anesthesia, neural pathway, nucleus accumbens, sevoflurane, ventral pallidum

## Abstract

**Background:**

General anesthesia has long been used in clinical practice, but its precise pharmacological effects on neural circuits are not fully understood. Recent investigations suggest that the sleep–wake system may play a role in the reversible loss of consciousness induced by general anesthetics. Studies in mice have shown that microinjection of dopamine receptor 1 (D1R) agonists into the nucleus accumbens (NAc) promotes recovery from isoflurane anesthesia, while microinjection of D1R antagonists has the opposite effect. Furthermore, during the induction and maintenance of sevoflurane anesthesia, there is a significant decrease in extracellular dopamine levels in the NAc, which subsequently increases during the recovery period. These findings suggest the involvement of the NAc in the regulation of general anesthesia. However, the specific role of D1R‐expressing neurons in the NAc during general anesthesia and the downstream effect pathways are still not well understood.

**Methods:**

In order to analyze the impact of sevoflurane anesthesia on NAc^D1R^ neurons and the NAc^D1R^‐VP pathway, this study employed calcium fiber photometry to investigate alterations in the fluorescence intensity of calcium signals in dopamine D1‐receptor‐expressing neurons located in the nucleus accumbens (NAc^D1R^ neurons) and the NAc^D1R^‐VP pathway during sevoflurane anesthesia. Subsequently, optogenetic techniques were utilized to activate or inhibit NAc^D1R^ neurons and their synaptic terminals in the ventral pallidum (VP), aiming to elucidate the role of NAc^D1R^ neurons and the NAc^D1R^‐VP pathway in sevoflurane anesthesia. These experiments were supplemented with electroencephalogram (EEG) recordings and behavioral tests. Lastly, a genetically‐encoded fluorescent sensor was employed to observe changes in extracellular GABA neurotransmitters in the VP during sevoflurane anesthesia.

**Results:**

Our findings revealed that sevoflurane administration led to the inhibition of NAc^D1R^ neuron population activity, as well as their connections within the ventral pallidum (VP). We also observed a reversible reduction in extracellular GABA levels in the VP during both the induction and emergence phases of sevoflurane anesthesia. Additionally, the optogenetic activation of NAc^D1R^ neurons and their synaptic terminals in the VP resulted in a promotion of wakefulness during sevoflurane anesthesia, accompanied by a decrease in EEG slow wave activity and burst suppression rate. Conversely, the optogenetic inhibition of the NAc^D1R^‐VP pathway exerted opposite effects.

**Conclusion:**

The NAc^D1R^‐VP pathway serves as a crucial downstream pathway of NAc^D1R^ neurons, playing a significant role in regulating arousal during sevoflurane anesthesia. Importantly, this pathway appears to be associated with the release of GABA neurotransmitters from VP cells.

## Graphical abstract

The underlying mechanism of general anesthesia remains unclear. In this study, the optogenetic was used as the major method to investigate the role of Dopamine D1‐receptor‐expressing neurons in the nucleus accumbens (NAc^D1R^) and their downstream projection in sevoflurane anesthesia. It found that activation of NAc^D1R^ neurons and the terminal of NAc^D1R^ neurons in the ventral pallidum (VP) facilitate the emergence from sevoflurane anesthesia, which suggests that NAc^D1R^ neurons play a significant role in regulating sevoflurane anesthesia through the downstream target VP.

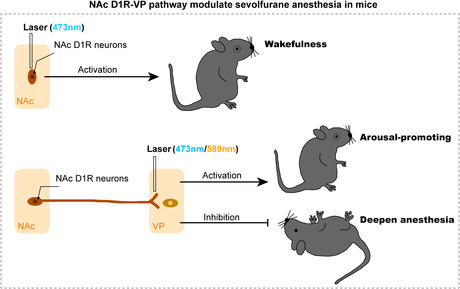

## 1 INTRODUCTION

Since its discovery by William T.G., nearly 180 years ago, general anesthesia has seen widespread use in the field of clinical practice. In 1846, Morton pioneered the use of ether as an anesthetic at the Massachusetts General Hospital. On the other hand, the mechanism that underlies the unconsciousness that is brought on by anesthetics is not well understood.^1^ As a result of recent research, there is mounting evidence to suggest that the endogenous sleep and arousal pathways in the brain may play a role in the regulation of general anesthesia.^2^, ^3^

The nucleus accumbens (NAc), located in the ventral striatum, is a crucial structure of the basal ganglia, comprising 95% of GABAergic medium spiny neurons (MSN) and the remaining consisting of cholinergic neurons and GABAergic interneurons.^4^ MSN in NAc can be subdivided further into two main subtypes: dopamine D1 receptor‐expressing MSN, also known as D1‐MSN, and dopamine D2 receptor‐expressing MSN (D2‐MSN).^4^, ^5^ Even though NAc is best known for its function as a component of the mesocortical limbic circuit, which is responsible for the control of the reward system, new research has shown that it also plays a crucial role in the regulation of the sleep–wake cycle.^4^, ^6^

In our previous study, we showed that the VTA^DA^‐NAc pathway and extracellular dopamine levels in the NAc are involved in regulating sevoflurane anesthesia.^7^ Other studies have also demonstrated that the NAc has a role in the regulation of general anesthesia through the activation of D1R^8^, ^9^ as well as the optogenetic activation of NAc^D1R^ neurons, which helps facilitate recovery from sevoflurane anesthesia.^10^ Nevertheless, the particular mechanisms that are at play are not yet completely known. The NAc has reciprocal connections with various arousal/sleep‐related structures such as the VTA, SN, LH, VLPO, and VP,^11^, ^12^, ^13^ but it remains to be determined how these downstream pathways of the NAc are involved in the mechanism of general anesthesia.

The ventral pallidum (VP), which is a component of the basal ganglia, is located in the ventral section of the anterior commissure. About 80 percent of the VP is made up of neurons that are GABAergic.^14^ Studies using a technique called retrograde tracing have shown that the nucleus accumbens (NAc) is the principal source of innervation for the ventral pallidum (VP).^15^, ^16^ Microinjection of a GABAA receptor agonist into the VP has been shown to promote sleep in mice, while an antagonist promotes wakefulness.^17^ Oishi et al. have discovered that optogenetic activation of the terminals of NAc neurons in the VP can reduce the firing rate of VP neurons and elicit inhibitory postsynaptic currents in those neurons. However, these effects were not observed in other downstream projection nuclei of the NAc, such as the lateral hypothalamus (LH), ventral tegmental area (VTA), and tuberomammillary nucleus (TMN).^18^ The findings imply that the NAc and VP have a close functional and synaptic link in the process of regulating sleep and wakefulness. On the other hand, it is currently uncertain whether or not this relationship plays a role in the modulation of consciousness under general anesthesia. In this study, we utilized c‐Fos staining, calcium fiber photometry, transmitter probes, optogenetics, and behavioral and EEG analyses to investigate the role of the NAc^D1R^‐VP pathway in sevoflurane anesthesia. To lay the groundwork for future study into the mechanisms that underlie general anesthesia by developing a conceptual framework.

## 2 MATERIALS AND METHODS

### 2.1 Animals

The guidelines provided in the Guide for the Care and Use of Laboratory Animals in China (No. 14924, 2001) were strictly followed throughout the course of this research project, and the Animal Care and Use Committees at Zunyi Medical University gave their stamp of approval. Adult male mice, including D1R‐Cre mice and C57BL/6J mice, weighing 22–28 g and aged 8–10 weeks, were utilized. Mice were kept in SPF standard chambers with a light/dark cycle of 12 h on and 12 h off. The temperature was kept at 23°C with a relative humidity of 55% and 2%, and they had free access to food and drink at all times. The experimental groups were assigned using a random selection process.

### 2.2 Drugs

RWD Life Science was the vendor for the purchase of sevoflurane and isoflurane. (Shenzhen, China). The dopamine D1R/DRD1 antibody (NBP2‐16213) and Mouse Monoclonal c‐Fos antibody (NBP2‐50037) were purchased from Novus Corporation (United States). The secondary antibodies consisted of goat anti‐rabbit antibodies that had been conjugated to Alexa 488 (ab150077, Abcam), goat anti‐rabbit antibodies that had been conjugated to Alexa 594 (ab150080, Abcam), and goat anti‐mouse antibodies that had been conjugated to Alexa 594 (ab150116, Abcam).

### 2.3 Virus injection

The animals were put under the influence of 1.4% isoflurane in oxygen (O2) at a flow rate of 1 L/min, and then they were immobilized in a stereotaxic frame (RWD Life Science). This was done after confirming that there was no longer a pain reaction or righting reflex. Erythromycin eye ointment was applied to protect the eyes, and the hair on the head was shaved using an electric hair shaver. To provide local anesthetic, lidocaine at a concentration of one percent was injected subcutaneously before exposing the surface of the skull. The head was readjusted in such a way that the bregma and lambda were brought into horizontal alignment with one another. Above the target locations, very small craniotomy holes, measuring between 300 and 500 um, were created. Using a microsyringe pump, the following adeno‐associated viruses were injected into the NAc (anterior–posterior [AP]: +1.7 mm, medial‐lateral [ML]: +0.6 mm, and dorsal‐ventral [DV]: −4.3 mm) or VP (anterior–posterior [AP]: +0.14 mm, ML: +0.6 mm, DV: −5.2 mm): Cre‐inducible GCaMP (rAAV2/9‐hSyn‐DIO‐GCaMP6s, PT‐0091; rAAV2/9‐hSyn‐GCaMP6s, PT‐0145), iGABA (rAAV9‐hSyn‐iGABA, PT1301), optogenetic (rAAV2/9‐EF1a‐DIO‐ChR2‐mCherry, PT‐0002; rAAV2/9‐EF1a‐DIO‐eNpHR‐mCherry, PT‐0007), and mCherry (rAAVEf1a‐DIO‐mCherry‐WPRE‐pA, PT‐0013), according to the mouse brain atlas (George Paxinos and Keith B. J. Franklin, second edition). Following the administration of the medication, the glass pipette remained in its position for the subsequent 10 min to allow for diffusion, after which it was gradually removed. Optic fibers were implanted unilaterally over the NAc (anterior–posterior [AP]: +1.7 mm, medial‐lateral [ML]: +0.6 mm, and dorsal‐ventral [DV]: −4.1 mm) or VP (AP: +0.14 mm, ML: +0.6 mm, DV: −4.9 mm), while mice used for optogenetic experiments were implanted with cortical electroencephalogram (EEG) electrodes and secured with four skull screws and dental cement. Before beginning any electrophysiological or behavioral research, animals were given at least 3 weeks to recuperate before the tests. Animals that had viral infection sites and fiber placements in the NAc or VP that could be independently verified were considered for inclusion in the study.

### 2.4 Fiber photometry

A fiber photometry system (ThinkerTech Nanjing Bioscience) was utilized to record changes in Ca^2+^ signals in mice. This system was equipped with a 480‐nm excitation LED (3W, CREE), a dichroic mirror (DCC3420M; Thorlabs), and a multifunction data collecting program (ThinkerTech Nanjing Bioscience Inc.). To transmit the light between the system and the implanted optical fiber, optical fibers manufactured by Newton Inc. in China and integrated with an optical diverter manufactured by Doric Lenses were employed. The Ca^2+^ signals were recorded as a baseline during the awake state, and then the mice were anesthetized with 2.4% sevoflurane to record changes in Ca^2+^ signals during anesthesia and recovery. The data from the fiber photometry were analyzed using MATLAB 2016a (MathWorks, Cambridge, United States), and the fluorescence changes (Δ*F*/*F*) were computed as (*F* − *F*0)/*F*0, where *F* is the signal from the test fluorescence and *F*0 is the signal from the baseline.

### 2.5 Monitoring GABA transmitters release

In this study, we utilized genetically encoded GABA sensors called GRABiGABA that respond to low concentrations of GABA neurotransmitter in the VP. These sensors were produced by linking conformationally sensitive circular‐permutated EGFP (cpEGFP) to particular GABA receptors, and they were expressed in the ventral paraventricular nucleus (VP) of C57BL/6 mice using adeno‐associated virus. (AAV).^19^ Changes in GABA concentration were reflected by changes in fluorescence intensity, which were measured with a multichannel fiber photometry system. The recording protocol was the same as that used for calcium fiber photometry. Due to the high sensitivity of the GRABiGABA sensors, it was possible to detect nanomolar as well as micromolar amounts of the neurotransmitter GABA.

### 2.6 Behavioral tests

Loss of righting reflex (LORR) and recovery of righting reflex (RORR) periods in mice are frequently employed as standardized indices for measuring the amount of time required for general anesthesia to be induced as well as the amount of time required to emerge from it.

In this study, we induced anesthesia in mice by placing them in an anesthesia chamber (10 × 20 × 15 cm) that had been allowed to adapt for 10 min. Subsequently, the mice were induced and maintained using 2.4% sevoflurane with 100% O2 at a flow rate of 1.5 L/min. A Vamos anesthetic monitor was utilized in order to track the amount of sevoflurane present in the operating room during the procedure. (Drager Company). The time that was recorded as the LORR was the amount of time that had passed since the beginning of the sevoflurane administration. The time that was recorded as the RORR was the amount of time that had passed after the end of the sevoflurane infusion. For the optogenetic experiments, we applied optical stimulation to NAcD1R neurons and their terminals, using pulses of 473 nm light with a 10 ms width at 20 Hz during the LORR period and pulses of 589 nm light with a 10 ms width at 10 Hz during the RORR period. Using an optical power meter, the optical power at the end of the fiber was calibrated to be between 10 and 15 milliwatts. (PM100D, Thorlabs). The LORR, RORR, and EEG were all recorded while the patient was under the influence of sevoflurane. Following the completion of the trials, each mouse was put through an immunofluorescence test to confirm the presence of the virus and the accuracy of the transfection.

### 2.7 EEG recording and spectral analysis

EEG signals were collected using a multichannel signal acquisition system (Apolo, Bio‐Signal Technologies) with a filter range of 0.1–300 Hz. Recordings were taken for 5 min prior to induction and continuously during the 20‐min sevoflurane anesthesia period until recovery. To further observe the effect of transient activation of neurons and pathways on sevoflurane anesthesia, after a 5‐min baseline EEG recording, 2.4% sevoflurane was originally administered for induction along with oxygen at 1.5 L/min. Then 10 min after sevoflurane inhalation, when stable BSR was present, mice were given 20 Hz blue light stimulation for 1 min, followed by inhalation anesthesia for another 10 min, and EEG changes were recorded. A power spectrum analysis was carried out on the EEG data, with the relative power in the *δ*, *θ*, *α*, *β*, and *γ* frequency bands is obtained by calculating the average signal power throughout each band and dividing it by the total power from 1 to 60 Hz, as was detailed in the prior paragraphs.^20^, ^21^ Burst suppression rate (BSR) was analyzed using an improved method in MATLAB 2016a (MathWorks) for the 20‐min period prior to the cessation of sevoflurane. Spectrograms were constructed using multitaper methods from the Chronux toolbox in MATLAB 2016a (MathWorks). EEG recordings were taken at least 5 days after behavioral testing to allow for recovery from anesthesia.

### 2.8 Histological verification

Isoflurane (1.4% concentration) and a subcutaneous injection of lidocaine (2% concentration) were used to induce general and local anesthesia, respectively, in mice. Following deep anesthesia, mice were transcranially infused with 150 mL of PBS and then 50 mL of 4% PFA. After removal, the brains were placed in 4% PFA at 4°C for 24 h, after which they were transferred to 30% sucrose in PBS at the same temperature until they sank. To verify the injection sites with reference to the mouse brain atlas 22, the brains were coronally sectioned into 30‐um slices using a cryostat (CM1950; Leica).^22^ After exposing each brain segment to a solution of PBS containing 2.5% normal goat serum, 1.5% bovine serum albumin, and 0.1% TritonTM X‐100 for a period of 2 h while at room temperature, the sections were blocked. After that, they were incubated with a primary antibody (rabbit anti‐DRD1 antibody, diluted 1:200) in blocking solution for 12 h at 4°C, and then they were washed three times for 10 min each with PBS. After that, the tissue slices were treated with a secondary antibody (goat anti‐rabbit antibody that had been conjugated to Alexa 488/594 at a dilution of 1:1000) at room temperature for a period of 2 h. The slices were then mounted on glass slides and coated with mounting material following one more wash with PBS that lasted between 3 and 10 min. (Gold antifade reagent with DAPI, Life Technologies). The Olympus BX63 Virtual Microscopy System was utilized in order to get the immunostaining images.

### 2.9 Statistical analysis

The GraphPad Prism software package, version 6.0, was utilized in order to carry out the statistical analyses. (GraphPad Software Inc.). The normality of data distribution was assessed by the Shapiro–Wilk test. Paired Student's *t*‐tests were used to compare calcium signals and GABA neurotransmitter signals between pre‐ and post‐events periods, as well as the change in LORR and RORR times for optogenetic experiments within the same group (light‐on vs. light‐off). Unpaired LORR and RORR timings, as well as the percentages of the EEG bands, were compared between the groups using Student's *t*‐tests. The expression of c‐Fos among the three groups was analyzed by one‐way ANOVA. The data are shown with a mean and a standard error of the mean, and a *p* value of 0.05 was judged significant in every instance.

## 3 RESULTS

### 3.1 Sevoflurane anesthesia inhibits the population activities of NAc^D1R^ neurons

**FIGURE 1:**
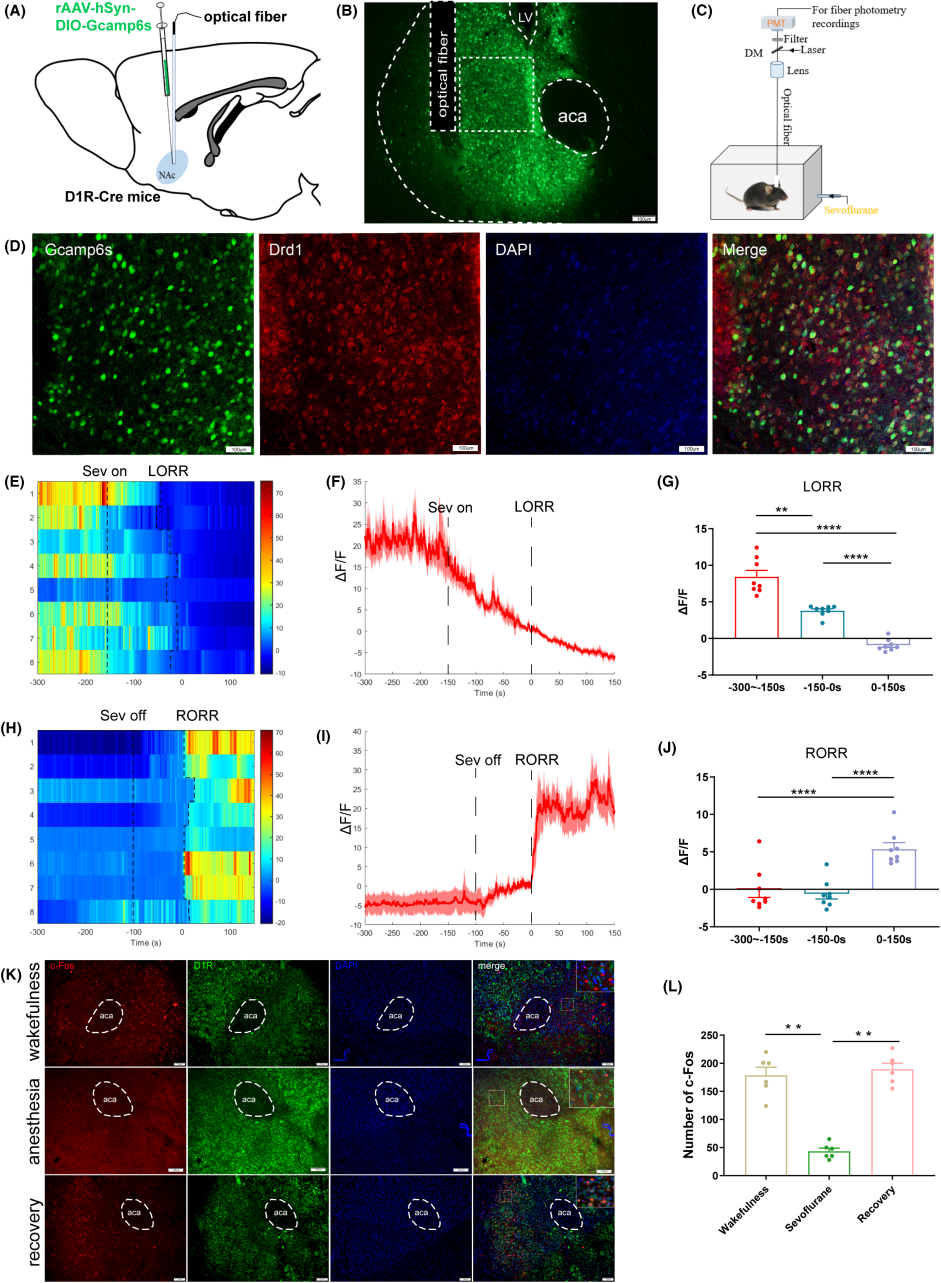
Phase‐dependent calcium alteration in NAcD1R neurons during sevoflurane anesthesia. (A) Schematic of establishing calcium signal recording model into the NAc in D1R‐Cre mice. (B) Representative image of the NAc^D1R^ expressing GCaMP6s and optical fiber implanting sites (scale bar = 100 mm). (C) Schematic diagram depicting fiber photometry recording during sevoflurane anesthesia in freely moving mice. (D) Expression of GCaMP6s in NAc^D1R^ neurons. Viral expression (GCaMP6s, green) in the NAc and colabeling with D1R expressing neurons (Drd1 immunofluorescence, red; DAPI, blue; scale bars: 100 mm). (E,H) Heat map illustrating the changes in the Ca2^+^ signals related to sevoflurane‐induced LORR and RORR. (F,I) Average calcium transients associated with LORR and RORR, mean (red trace) ± SEM (red shading). (G,J) Δ*F*/*F* represents the deviation in Ca2^+^ fluorescence from the baseline, which is the averaged Δ*F*/*F* between *t* = −300 s and −150 s. (K) Expression of c‐Fos in the NAcD1R neurons in the wakefulness state, sevoflurane anesthesia, and recovery from anesthesia in mice (scale bars: 100 mm, *n* = 6). (L) The average number of c‐Fos among the three groups. Data are presented as the mean ± SEM. *p < 0.05, **p < 0.01, ***p < 0.001, *n* = 8, by paired t‐test; c‐Fos expression statistic analyzed by one‐way ANOVA.

In order to investigate the levels of activity shown by NAc^D1R^ neurons, we started by looking at the expression of an immediate early gene called c‐Fos. This gene is well recognized as a trustworthy predictor of neuronal activity.^23^ Using wild‐type mice, we administered anesthesia with 2.4% sevoflurane and an oxygen flow rate of 1.0 L/min, after which the mice were sacrificed for immunostaining. Co‐staining of Fos and D1R revealed that the proportion of Fos‐positive D1R‐expressing neurons in the NAc decreased following 2 h of sevoflurane anesthesia (43.5 ± 5.506) compared to the wakefulness (178.7 ± 14.3) and recovery (189.7 ± 10.73) states. According to this observation, the activity of NAc^D1R^ neurons was considerably reduced by sevoflurane anesthesia (*p* < 0.01, *n* = 6; see Figure 1K,L).

In order to further investigate the real‐time activity of NAc^D1R^ neurons during sevoflurane anesthesia, we stereotactically infused Cre‐dependent adeno‐associated virus (AAV) encoding the fluorescent calcium indicator GCaMP6s into the NAc of D1R‐Cre mice (Figure 1A). We then used fiber photometry to record changes in Ca^2+^ signals in vivo while the mice were under sevoflurane anesthesia. The majority of GCaMP6‐expressing cells were found to be positive for D1R after being stained with immunofluorescence (Figure 1B,D). During the process of inducing anesthesia with sevoflurane, we divided the analysis of Ca^2+^ signals into three periods: the wake period (−300 to −150 s), the induction period (−150 to 0 s), and the anesthesia period (0–150 s). Following the onset of sevoflurane, the calcium signal of NAc^D1R^ neurons began to decrease significantly, with the decrease starting slowly before the appearance of loss of righting reflex (LORR), and continuing to decrease and maintain at a low level after LORR (*p* < 0.01, *n* = 8; see Figure 1E–G). When the sevoflurane was turned off, the calcium signal gradually recovered and increased significantly after the return of righting reflex (RORR). In the recovery period, we analyzed three phases, including the anesthesia period (−300 to −150 s), the recovery period (−150 to 0 s), and the wake period (0–150 s). A robust increase in activity due to RORR was observed (*p* < 0.0001, *n* = 8; see Figure 1H–J). The findings presented here indicate that sevoflurane anesthesia has an effect on the activity of NAc^D1R^ neurons.

### 3.2 Activation of NAc neurons both delayed the establishment of sevoflurane anesthesia and aided the recovery from it

**FIGURE 2:**
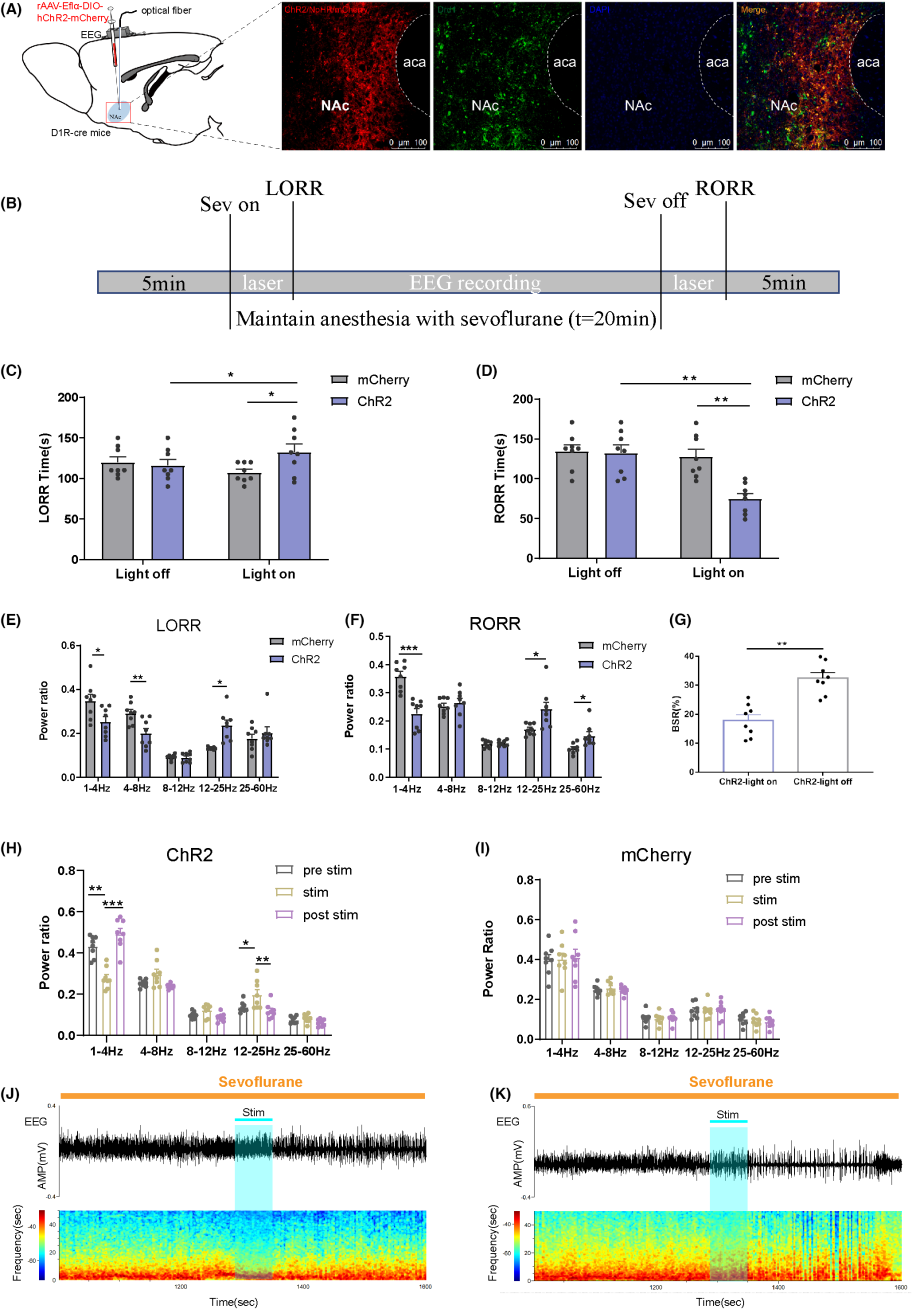
Optogenetic activation of NAc^D1R^ neurons facilitates arousal from sevoflurane anesthesia. (A) Diagram of the optogenetic virus injection and stimulation sites and the expression of virus (mCherry, red) in NAc^D1R^ neurons and colabeling with D1R (Drd1 immunofluorescence, green; scale bar: 200 mm). (B) Schematic of the experimental time‐course. (C,D) Optical activation of NAc^D1R^ neurons prolonged the induction time and shortened the emergence time from sevoflurane anesthesia. (E,F) Comparison of each Electroencephalogram (EEG) frequency band between the two groups during optogenetic activation of NAc^D1R^ neurons. (G) BSR during the maintenance of sevoflurane in ChR2‐light on or ChR2‐light off. (H,I) Relative EEG power before (gray), during (yellow), and after (purple) acute photostimulation at 20 Hz in ChR2 or mcherry mice. (J,K) Representative EEG traces and the corresponding heat map of the process in the two groups. Data are presented as the mean ± SEM. *p < 0.05; **p < 0.01, *n* = 8, by paired and unpaired t‐test.

We then investigated the role of NAc^D1R^ neurons using optogenetics, behavioral tests, and EEG recordings in D1R‐cre mice. Compared to the mCherry group, blue light stimulation at 20 Hz at the onset of sevoflurane administration prolonged the LORR time (132.75 ± 9.90 vs. 107.25 ± 4.19, *p* < 0.05), while optical activation of NAc^D1R^ neurons at the end of sevoflurane inhalation until RORR shortened the arousal time (74.87 ± 6.62 vs. 127.75 ± 9.51, *p* < 0.05). When compared to the light‐off control, laser stimulation within each group significantly lengthened the LORR time (132.79.9 vs. 116.257.1, *p* < 0.05) and considerably lowered the RORR time (87.414.2 vs. 132.59.7, *p* < 0.05; Figure 2C,D). Additionally, EEG recordings showed a significant difference in the power spectrum between the ChR2 and mCherry groups (Figure 2E,F). During the induction of sevoflurane anesthesia, the ChR2 group exhibited a decrease in δ waves (1–4 Hz) and θ waves (4–8 Hz) accompanied by an increase in β waves (12–25 Hz) compared to the mCherry group (*p* < 0.05). Conversely, during the emergence of RORR, there was a strikingly decreased ratio of δ bands and an increased ratio of β and γ (25–60 Hz) bands (*p* < 0.05) in the ChR2 group. The 20 Hz photostimulation of NAc^D1R^ neurons in ChR2 mice also resulted in a remarkable decrease in BSR (Figure 2G, *p* < 0.01). According to these findings, throughout both the LORR and RORR procedures, blue light had an attenuating effect on low‐frequency wave bands, while it had an amplifying effect on high‐frequency wave bands when blue light was applied to the ChR2 group. Furthermore, transient light stimulation (for 1 min) during burst inhibition led to a sharp decrease in δ bands and a significant increase in β bands (Figure 2H–K, *p* < 0.05). Overall, these results suggest that activation of NAc^D1R^ neurons plays a modulatory role in the processes of sevoflurane anesthesia induction and emergence.

### 3.3 Activities of NAc^D1R^‐VP pathway and neurons of VP in response to sevoflurane anesthesia

**FIGURE 3:**
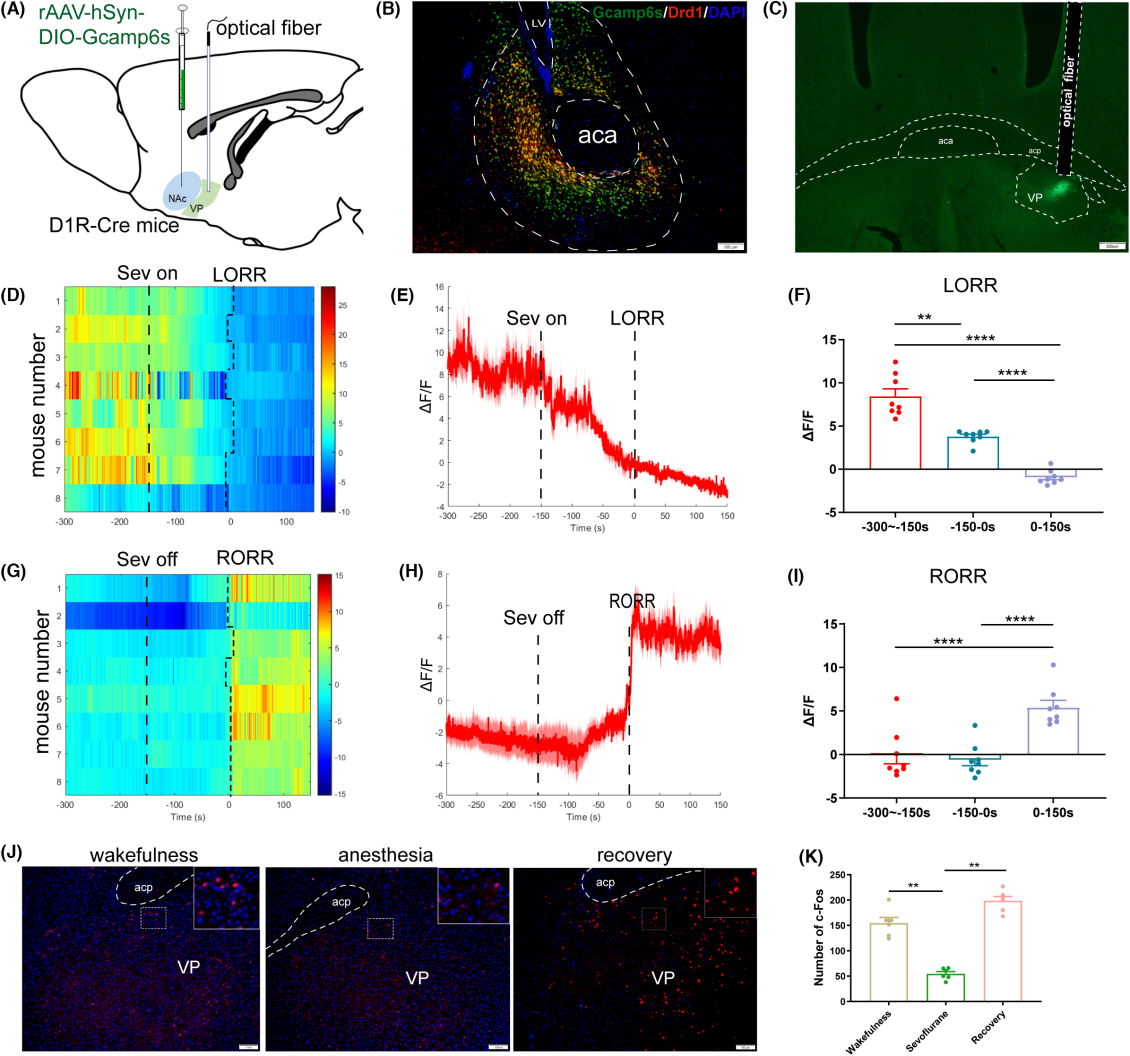
Sevoflurane anesthesia inhibited the activity of NAc^D1R^‐VP pathway and neurons in VP. (A) Schematic of establishing calcium signal recording model. (B) Expressing of Gcamp6s in NAc colabeling with NAc^D1R^ neurons (scale bars: 100 mm). (C) Expression of NAc^D1R^ neuron terminals in the VP (scale bars: 200 mm). (D,G) Heat map illustrating changes in the Ca2+ signals related to sevoflurane‐induced LORR and RORR. (E,H) Average calcium transients associated with loss of righting reflex (LORR) and recovery of righting reflex (RORR), mean (red trace) ± SEM (red shading). (F,I) Δ*F*/*F* represents the deviation in Ca2+ fluorescence from the baseline, which is the average. ΔF/F between *t* = −300 s and − 150 s. Data are presented as the mean ± SEM. *p < 0.05; **p < 0.01; ***p < 0.001, n = 8, by paired *t*‐test. (J) Expression of c‐Fos in the VP during the wakefulness state, sevoflurane anesthesia, and recovery from anesthesia in mice (scale bars: 100 mm). (K) An average number of c‐Fos‐immunopositive neurons. One‐way ANOVA followed by post hoc Bonferroni's test. *n* = 6, *p < 0.05, **p < 0.01.

In order to investigate the real‐time activity of the NAc^D1R^ ‐VP pathway during sevoflurane anesthesia, we utilized cre‐dependent rAAV‐hSyn‐DIO‐GCaMP6s to label the NAc of D1R‐cre mice, placed an optical fiber cannula above the VP, and utilized fiber photometry to record in vivo changes in Ca^2+^ signals during isoflurane anesthesia (Figure 3A–C). We analyzed the Ca^2+^ signals during three periods of the induction of sevoflurane anesthesia: the wake period (−300 to −150 s), induction period (−150 to 0 s), and anesthesia period (0–150 s). After sevoflurane‐induced LORR, the Ca^2+^ signals significantly decreased (*p* < 0.001, *n* = 8), and remained at a low level even while the mice were under the influence of sevoflurane anesthesia (*p* = 0.001, *n* = 8; Figure 3D–F). During the recovery period, we analyzed three time periods, including the anesthesia period (−300 to −150 s), recovery period (−150 to 0 s), and wake period (0–150 s). A robust increase due to RORR was observed (*p* < 0.01, *n* = 8; Figure 3G–I). It appears from these observations that the use of sevoflurane for anesthesia reduces the activity of the NAc^D1R^ ‐VP.

In addition, we used c‐Fos labeling to investigate the connection between the neuronal activity of the ventral posterior (VP) region and the influence of sevoflurane anesthesia. Our data demonstrated that c‐Fos expression decreased after 2‐h of sevoflurane anesthesia (54.50 ± 4.50) compared to the wakefulness (154.50 ± 11.19) and recovery (198.33 ± 8.68) states (*p* < 0.01, *n* = 6, Figure 3J,K). This result suggests that neuronal activity in the VP could be associated with the stages of sevoflurane anesthesia.

### 3.4 Activation or inhibition of GABAergic terminals of NAc^D1R^ neurons in the VP by optical means modulate the recovery from the effects of sevoflurane anesthesia

**FIGURE 4:**
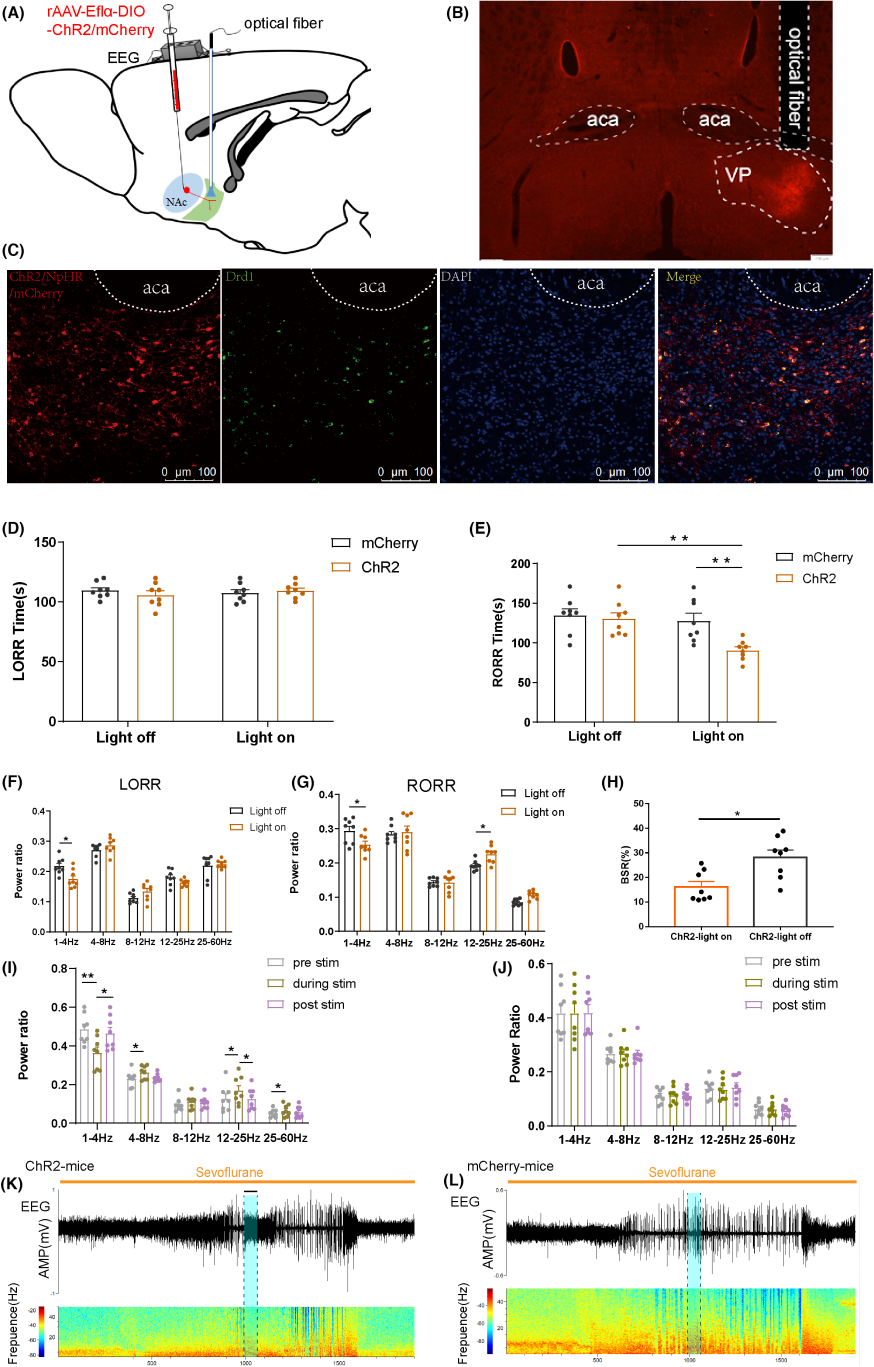
Optogenetic activation of the NAc^D1R^ ‐VP pathway modulates the process of sevoflurane anesthesia. (A) Schematic of optogenetic virus injection and stimulation sites. (B) Expression of NAc^D1R^ neurons terminal in the NAc. (C) Expression of ChR2/NpHR/mCherry (red) and D1R immunofluorescence (green) in the NAc. (D,E) Optical stimulation of NAc^D1R^ GABAergic projections in the VP reduced the emergence time but not the induction time. (F,G) Optogenetic activation of axons emanating from NAc^D1R^ neurons in VP mediated the band distribution of EEG power during both the LORR and RORR process. (H) BSR during the maintenance of sevoflurane in ChR2‐light on or ChR2‐light off. (I,J) Relative EEG power before (gray), during (yellow), and after (purple) acute photostimulation at 20 Hz in ChR2 mice. (K,L) Representative EEG traces and heat maps from each group. Data are presented as the mean ± SEM. *p < 0.05, **p < 0.01, *n* = 8, by paired and unpaired *t*‐test.

**FIGURE 5:**
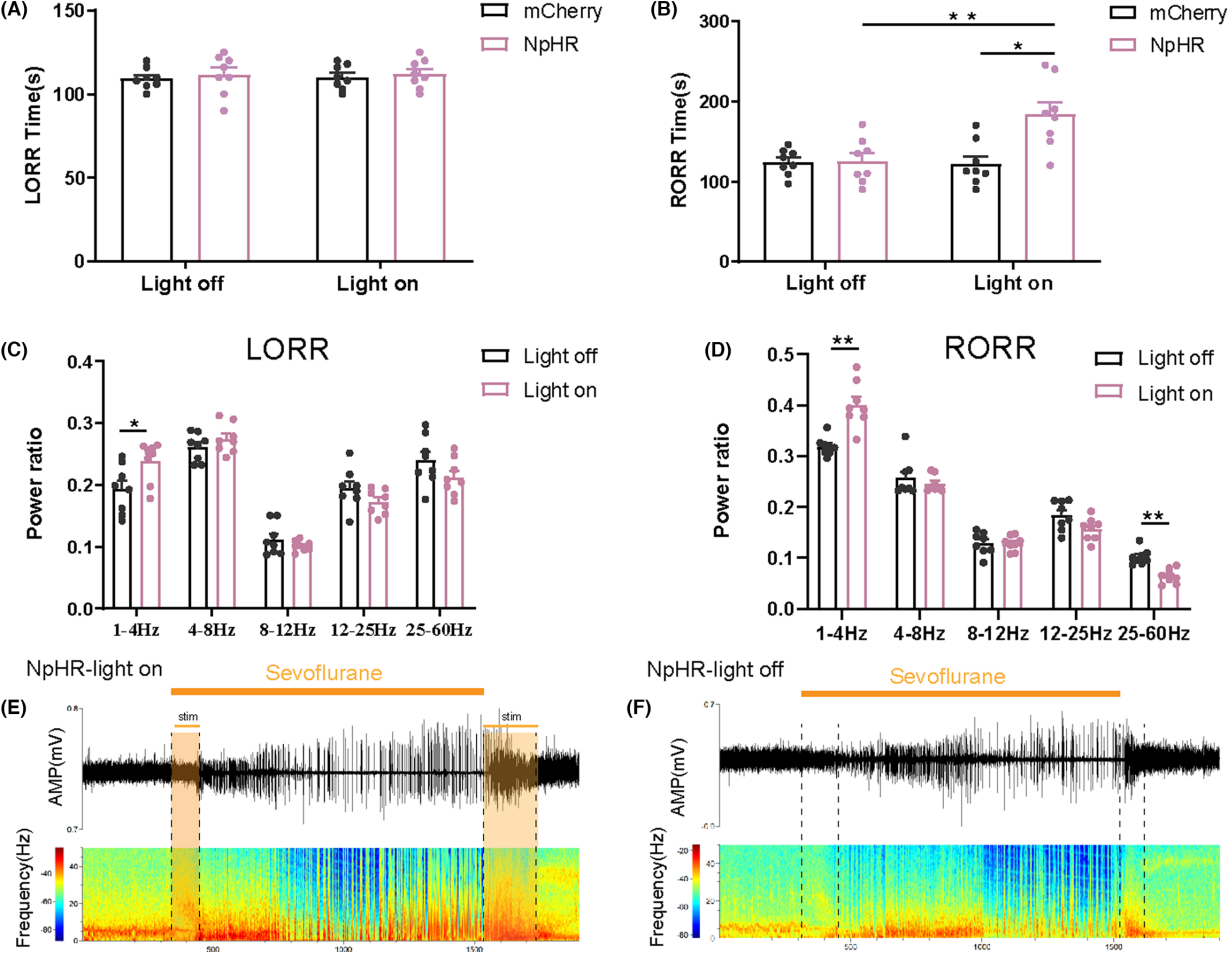
Optogenetic inhibition of NAc^D1R^ ‐VP pathway prolongs emergence from sevoflurane anesthesia. (A,B) Optogenetic inhibition of NAc^D1R^ GABAergic projections in the VP prolonged emergence from sevoflurane anesthesia, but did not affect induction time. (C,D) Optogenetic inhibition of axons emanating from NAc^D1R^ neurons in VP mediated the band distribution of EEG power during both the LORR and RORR process. (E,F) Representative EEG traces and heat maps from each group. Data are presented as the mean ± SEM. *p < 0.05, **p < 0.01, *n* = 8, by paired and unpaired *t*‐test.

To further investigate the involvement of the NAc^D1R^ ‐VP pathway in general anesthesia, we administered AAV‐DIO‐ChR2‐mCherry, AAV‐EF1a‐DIO‐NpHR‐mCherry, or AAV‐DIO‐mCherry to the NAc of D1R‐Cre mice and inserted a fiber optic probe above the VP at the same time. Behavioral observations revealed that stimulation of the NAc^D1R^ terminals in the VP of ChR2 mice at 20 Hz resulted in a significant emergence from anesthesia and decreased the RORR time (130.37 ± 7.74 vs. 90.50 ± 4.38, *p* < 0.01), while mCherry mice exhibited no behavioral responses during photostimulation (Figure 4D,E), and did not significantly affect the amount of time required for LORR (Figure 4D). Additionally, in ChR2 mice, rapid transitions from slow‐wave activity or a burst‐suppression mode to low‐voltage fast activity were elicited by photostimulation of the NAc^D1R^‐VP pathway at 20 Hz (Figure 4K), but not in the control group (Figure 4L). Compared with the mcherry group, EEG spectral analysis demonstrated that instantaneous photostimulation (for 60 s) of NAc^D1R^‐VP pathway at 20 Hz induced an obvious decrease in δ band and an increase in β and γ bands (Figure 4I,J). Photoinhibition of the NpHR group extended the RORR time (125.37 ± 9.76 vs. 183.75 ± 15.08, *p* < 0.01) but did not significantly affect the LORR time (Figure 5A,B). EEG analysis showed that in the ChR2 group, δ waves (1–4 Hz) decreased during the induction period of sevoflurane anesthesia compared to the mCherry group, whereas differences in other bands were not significant according to statistical analysis. During the awakening period, δ waves decreased significantly, while β and γ waves increased, and α and θ waves showed no significant difference (Figure 4F,G). Besides, activation of the NAc^D1R^‐VP pathway reduces BSR during sevoflurane anesthesia (Figure 4H). When compared to the control group, the NpHR group had an increase in the number of beta waves that occurred during the induction of sevoflurane anesthesia. During anesthesia recovery, δ waves increased significantly, and α, β, and γ waves decreased, while θ waves had no statistical significance (Figure 5C,D).

### 3.5 Changes in the extracellular concentration of GABA in VP in response to sevoflurane anesthesia

**FIGURE 6:**
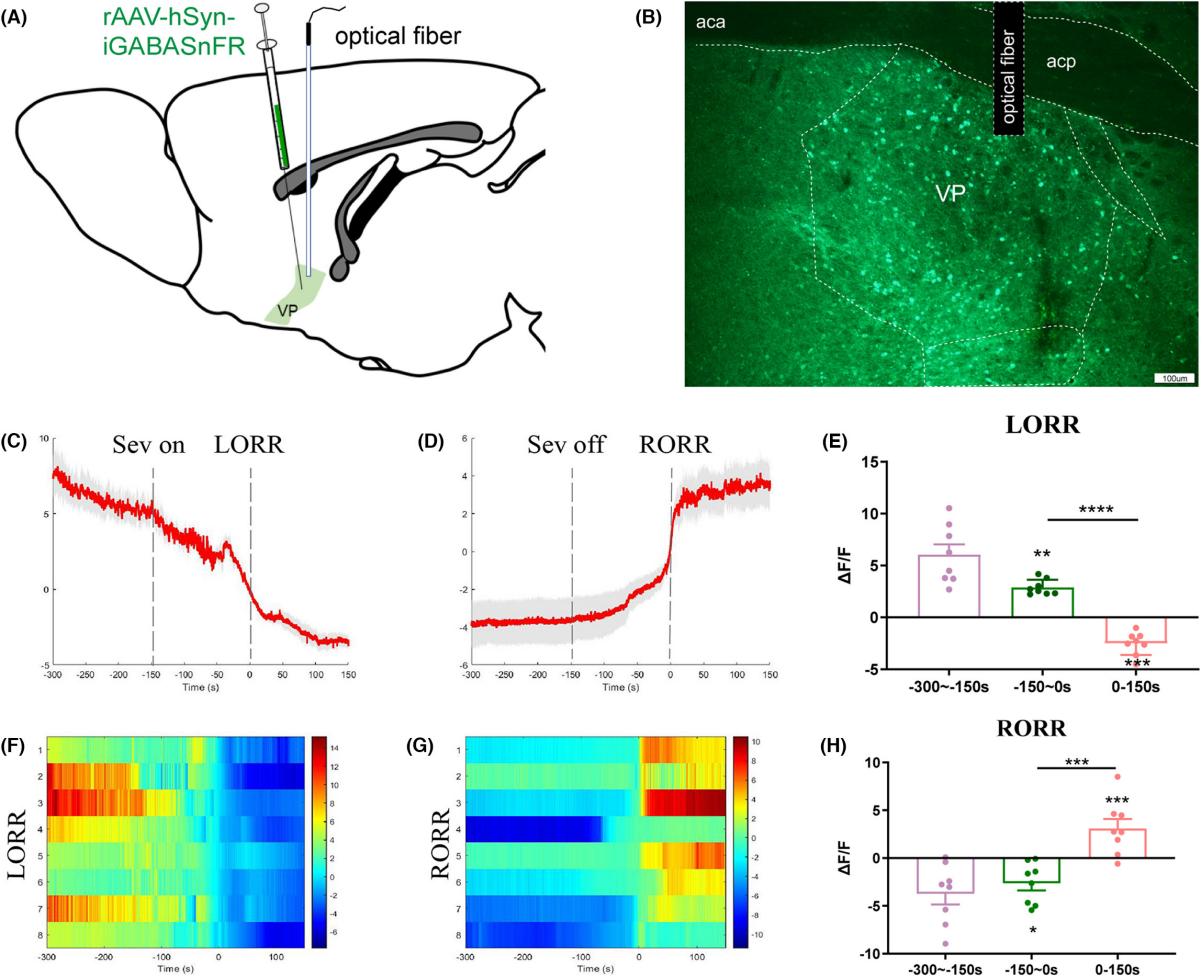
Dynamics of extracellular GABA in the VP in response to sevoflurane anesthesia. (A) Diagram of the sensor virus injection and recording sites. (B) Expression of the iGABASnFR virus in the VP area (scale bars: 100 mm). (C,D) Average GABA transmitters transients associated with LORR and RORR; mean (red trace) ± SEM (red shading). Note that the extracellular GABA fluorescence signals gradually decreased with LORR and increased sharply after RORR. (F,G) Heat map illustrating the changes in the GABA fluorescence signals related to sevoflurane‐induced LORR and RORR. (E,H) Δ*F*/*F* represents the deviation in the GABA transmitter signal from the baseline, which is the averaged Δ*F*/*F* between *t* = −300 s and − 150 s. Data are presented as the mean ± SEM. *p < 0.05; ***p < 0.001; ****p < 0.0001, *n* = 8, by paired *t*‐test.

To investigate the changes in extracellular GABA levels in the VP during sevoflurane anesthesia, we measured the dynamics of GABA concentration. Our findings indicate that during the induction of sevoflurane anesthesia, the extracellular GABA levels in the VP were significantly reduced compared to baseline. This suggests that the release of extracellular GABA in the VP is decreased during sevoflurane anesthesia. We also found that the GABA signals were significantly increased during RORR compared to anesthesia, indicating that sevoflurane modulates the release of extracellular GABA transmitter in the VP (Figure 6, *p* < 0.01).

## 4 DISCUSSION

This study aimed to investigate the role of NAc^D1R^ neurons and the NAc^D1R^ ‐VP pathway in sevoflurane anesthesia. Optogenetic activation of these neurons and pathways was found to boost behavioral arousal and cortical EEG activation in sevoflurane‐anesthetized mice. This finding indicates that they are capable of counteracting the anesthetic effects of sevoflurane. The study also revealed that sevoflurane inhibits the level of extracellular GABA in VP, suggesting that GABAergic transmission in VP is involved in sevoflurane‐induced loss of consciousness.

We used real‐time in vivo fiber photometry to explore the changes in calcium signals of NAc^D1R^ neurons during sevoflurane‐induced loss of righting reflex (LORR) and subsequent recovery of righting reflex (RORR), which was consistent with the findings of Huang and colleagues. LORR is for loss of righting reflex, while RORR stands for recovery of righting reflex.^10^ Our previous study demonstrated that the calcium signals of NAc neurons decreased during the induction of propofol anesthesia and subsequently recovered during the emergence from anesthesia in rats, and this effect was mediated by D1R signaling.^8^ Furthermore, optogenetic activation of NAc^D1R^ neurons facilitated emergence from anesthesia and increased the power of β and γ frequency bands in ChR2^+^ mice during sevoflurane anesthesia. Consistent with our results, previous studies demonstrated that chemo‐genetic or optogenetic activation of NAc^D1R^ neurons prolonged the induction time but shortened the emergence time of sevoflurane anesthesia in mice, whereas inhibition of NAc^D1R^ neurons produced opposite effects.^10^

According to our findings, NAc^D1R^ neurons appear to play a significant part in the regulation of sleep–wake cycles as well as general anesthesia. NAc^D1R^ neurons project to various brain regions involved in the regulation of sleep–wake, including VTA, VP, LH, VLPO, and others, and NAc provides the primary GABAergic inputs to VP.^12^, ^13^, ^15^ Previous studies have shown that brief optic stimulation of NAc^D1R^ neuron terminals in the VTA or LH can induce an arousal effect in mice, immediately transitioning them from NREM to wakefulness, and long‐term stimulation of these terminals increases wakefulness time.^6^ In addition, optic stimulation of the terminals of NAc^A2AR^ neurons in VP reduces the firing rate of VP neurons and induces inhibitory postsynaptic currents, this would imply that the NAc‐VP pathway has a role in the control of sleep and wakefulness. According to the findings of this study, the NAc^D1R^‐VP pathway is also engaged in the process of regulating consciousness while the patient is under general anesthesia.

Previous studies have indicated that optogenetic activation of VP^GABA^‐VTA and VP^GABA^‐LH projections in vivo can promote the transition from NREM to arousal via disinhibition of VTA‐TH^+^ cells and orexin neurons in LH.^24^, ^25^ Based on these findings, we hypothesize that the NAc^D1R^‐VP circuit involved in arousal regulation may partially achieve this effect by inhibiting VP GABAergic interneurons, resulting in the disinhibition of downstream nuclei. Therefore, our data suggest that the NAc^D1R^ ‐VP pathway may serve as an effective central neural circuit promoting arousal from general anesthesia.

Furthermore, our experiments showed that activation or inhibition of the NAc^D1R^ ‐VP pathway did not affect the induction time of anesthesia. This may be due to the fact that the NAc^D1R^‐VP pathway is primarily involved in regulating the recovery phase of anesthesia rather than the induction phase. Many brain regions have been identified to only participate in the regulation of consciousness during the recovery phase of general anesthesia, such as the basal ganglia and DRN‐5HT neurons.^26^, ^27^, ^28^ For example, a study demonstrated that microinjection of a D1R agonist into the basal ganglia promoted recovery from propofol anesthesia in rats, but did not affect the induction time.^29^ Similarly, activation of DRN‐5HT neurons using optogenetics and chemo genetics promoted recovery from isoflurane anesthesia but did not affect the induction time of anesthesia.^27^ These studies suggest that the mechanisms underlying the induction and recovery of general anesthesia are not regulated by a single neural network in a reversible process. Other neural circuits may also mediate the wakefulness induced by the activation of NAc^D1R^ neurons, such as the downstream wake‐promoting areas like VTA or LH.

During our study, we observed that the cortical EEG during induction did not correlate with the behavioral recovery of righting reflex. Previous investigations have indicated that there is a disconnect between brain activity and the formation of behavioral patterns.^30^, ^31^ A case in point is that Yin et al. used optogenetics to inhibit the VP^GABA^‐LH projection pathway, which promoted cortical arousal, but did not alter the time to RORR in mice under isoflurane anesthesia.^32^ The dissociation between cortical EEG and behavior may be attributed to the functional differences between different brain regions. However, future studies are needed to further investigate and interpret this inconsistency.

Our study revealed a significant decrease in c‐Fos expression within the ventral pallidum (VP) during sevoflurane anesthesia compared to wakefulness and the recovery period. Based on these findings, sevoflurane appears to be able to reduce the activity of VP neurons. A previous study has shown that the VP is predominantly made up of GABAergic neurons. Furthermore, during the transition from non‐rapid eye movement sleep (NREMs) to either wakefulness or rapid eye movement sleep (REMs), the population activity of VP GABAergic neurons increases.^24^ In addition, research has revealed that chemogenetic and optogenetic activation of VP GABAergic neurons may play a role in the process of arousal initiation and/or maintenance.^24^

Real‐time monitoring of GABA transmitters with GRAB sensors was used to investigate the effect of GABAergic projection from NAc^D1R^ neurons on VP neurons during sevoflurane anesthesia. The results showed that GABA transmitter levels began to decline upon sevoflurane administration, remained low throughout the anesthesia period, and increased during the anesthesia‐to‐wake transition. As GABA is an inhibitory transmitter, these data suggest that VP extracellular GABA transmitters are crucial for inducing recovery from sevoflurane anesthesia. This finding is consistent with previous studies demonstrating that activation of GABAergic neurons in VP and basal forebrain induces arousal.^24^, ^33^ Collectively, these results indicate that NAc^D1R^ neurons play a wake‐promoting role in sevoflurane anesthesia by regulating the release of GABA in their downstream VP.

In conclusion, the NAc^D1R^ ‐VP pathway represents a promising central neural pathway that promotes recovery from sevoflurane anesthesia, and as a result, may be a viable focus for future investigations on the effects of general anesthesia on consciousness changes. Furthermore, this study provides a new theoretical basis for understanding the mechanism of general anesthesia, as well, to decrease the incidence of issues brought on by delayed waking, reduce the amount of time needed to recover from general anesthesia and elevate the standard of care provided by clinical anesthesia.

However, the study has several limitations. First, the specific types of neurons that receive NAc^D1R^ neuron projections in VP are yet to be determined. Second, the electrophysiological mechanism underlying the regulation of sevoflurane anesthesia by the NAc^D1R^ ‐VP pathway remains unknown. Finally, it is unclear whether NAc^D1R^ neurons regulate general anesthesia through other projection pathways, and further research is needed to explore this possibility.

## AUTHOR CONTRIBUTIONS

J.Z. conceived and performed the experiments, as well as analyzed the data, completed the figures, and wrote the manuscript. Y.‐T.P. performed the experiments, analyzed the data, and wrote the manuscript. C.‐X.L., and Y.Z. analyzed the data and revised the manuscript. X.‐L.L., C.‐D.Y., and W.‐Y.S. collected the data. Y.Z. conceived the experiments, as well as revised the manuscript.

## FUNDING INFORMATION

This work was supported by the National Natural Science Foundation of China (81860204), Basic Research Project of the Department of Science and Technology of Guizhou Province (Qiankehe basic ZK [2022] General 649, Qiankehe basic ZK [2022] General 597, Famous Clinical Doctor Program ([2021]002) of the Zunyi Medical University), and Guizhou High‐Level Innovative Talent Training Program “Thousand” Level Talents Program.

## CONFLICT OF INTEREST STATEMENT

All authors have completed the ICMJE uniform disclosure form. The authors have no conflicts of interest to declare.

## Data Availability

The data that support the findings of this study are available on request from the corresponding author. The data are not publicly available due to privacy or ethical restrictions.
